# Health care service provision in Europe and regional diversity: a stochastic metafrontier approach

**DOI:** 10.1186/s13561-018-0195-5

**Published:** 2018-05-31

**Authors:** Katharina Schley

**Affiliations:** University of Goettingen, Humboldtallee 3, Göttingen, 37073 Germany

**Keywords:** Health production, Health efficiency, Stochastic frontier analysis, Metafrontier analysis

## Abstract

**■■■:**

In the last decades, demographic change coupled with new and expensive medical innovations have put most health care systems in developed countries under financial pressure. Therefore, ensuring efficient service provision is essential for a sustainable health care system. This paper investigates the performance of regional health care services in six West European countries between 2005 and 2014. We apply a stochastic metafrontier model to capture the different conditions in the health care systems in the countries within the European Union. By means of this approach, it is possible to detect performance differences in the European health care systems subject to different conditions and technologies relative to the potential technology available. The results indicate that regional deprivation plays a key role for the efficiency of health care provision. Furthermore, a pooled model which assumes a similar technology for all countries cannot sufficiently account for differences between countries. Surprisingly, the Scandinavian regions lag behind other regions with respect to the metafrontier.

**JEL Classification:**

C23, D61, I12, I18, R10

## Background

Demographic and social changes likely rise the burden of chronic diseases, like cancer, cardiovascular diseases, and diabetes. Along with new and expensive treatment options this puts the health care systems around the world under financial pressures. As a result of an increasing number of patients health care budgets in many countries inflate. Already, health expenditure grows more rapidly than the economy in many countries [7] comprising the sustainability of health care funding.

The increasing demand for new and innovative treatments and an increasing request for better value for money for patients at constant budgets raised the topic of *value based care* [31, 32]. In this sense, Porter [31] diagnoses a need to restructure the delivery of care to obtain sustainable health care budgets. Along these lines, the evaluation of the allocation and utilisation of medical resources is likely an important lever for the assessment of the performance of health care systems.

Several attempts have been made to analyse cross-country differences in the performance of health care services. A pioneer work is by Evans et al. [15]. The authors analyse the efficiency of 191 countries based on data provided by the World Health Organisation (WHO). Greene [19] challenged the work by Evans et al. [15] demonstrating that considerable unobserved heterogeneity due to cultural and economic characteristics leads to an underestimation of systematic health care differences. Accordingly, Greene [19] respecified the model introducing fixed effects parameters. His ‘true’ fixed effects model is able to distinguish between unobservable cross-country heterogeneity not associated with inefficiency and inefficiency itself. For the European Union, few studies have analysed the performance of health care systems. For instance, Asandului et al. [2] analyse the efficiency of a cross-section of 30 European countries by means of a Data Envelopment Analysis. They show that some countries lie on the production frontier while most are underneath. The analysis of the regional variation of health outcomes and performance of health care services at the national level has recently gained increasing interest ([11, 17, 20, 29, 33], for example).

So far, very little attention has been paid to the evaluation of performance differences in a cross-country context at the regional level. This would increase the understanding of underlying factors of cross-country performance differences as many countries are faced by regional variations in health outcomes and the availability of medical infrastructure. Even though, national governments decide in general on health policy measures municipalities and other regional level bodies are responsible for the provision of medical services further highlighting the need for a regional level analysis. Furthermore, potential sources of inefficiency such as an over or under use of services are likely located at the regional level [17]. Against this background, the aim of this study is to evaluate the efficiency and performance of health care services at the regional level in (Western) Europe. For this purpose, we apply a stochastic metafrontier model [4, 28]. The metafrontier model is essentially a two step approach. In the first step, country specific frontiers are estimated. In a second step, the metafrontier production function, which is a deterministic parametric function enveloping the individual frontiers, is calculated. This approach has the advantage that cross-country differences in the utilised technology are taken into account. The general frontier model assumes that all producers use the same technology. Based on this, one would assume that in the health care setting all health care systems are subject to the same rules and regulations. Bos and Schmiedel [5] relate this to the benchmark paradox. In the European framework, this is not the case as the design of the European health care systems is heterogeneous. In general, though, the health care systems in the European Union have access to similar health care inputs and the same technology which is taken into account by means of the metafrontier. Moreover, it is possible to distinguish between the regional efficiency in relation to the country’s own frontier and the metafrontier. For the analysis, we combine regional administrative information from six European countries (Austria, France, Germany, Italy, Scandinavia and Spain) for an extended time frame covering the period from 2005 to 2014.

To preview some results, we find that regional deprivation plays a key role for the efficiency of health care provision. Furthermore, we show that the efficiency scores of the health care systems in a pooled model, which assumes a common technology for all countries, differ from the efficiency scores with respect to the metafrontier.

The next section describes the stochastic frontier model and the metafrontier approach and introduces the data. Empirical results are discussed in the Section Results and discussion. The last section concludes. The appendix provides a detailed description of the data (Appendix Appendix A: Data description).

## Methods

In this section, we describe the empirical model to assess the stochastic health frontier and metafrontier approach. Moreover, we introduce the data and the variables.

### The stochastic frontier model

The *stochastic frontier analysis* (SFA) has become a common approach to asses production potentials and inefficiencies in the production of goods and services in, for example, farms, firms and hospitals. In contrast to standard non-parametric approaches which treat any deviation from a production function as inefficiency [8], the parametric SFA model allows to differentiate random deviations from the efficient frontier and inefficiency. We base the formalisation of a one-step SFA model on an approach proposed by Wang and Schmidt [37]. By quantifying production frontier parameters and exogenous influences on inefficiency simultaneously, we avoid the risk of biased estimation results inherent in two-step approaches based on estimated efficiency scores [37].

### The health frontier

In order to evaluate performance differences in the regional provision of health care services, we define the European regions as producers of health (DMUs). In a strict sense, the regions themselves do not convert inputs into outputs. However, they can be considered as (health) producers in a wider sense as they provide a framework for the provision of health care services [21]. The pooled SFA model for health production in region *i* at time *t* reads as

1 $$\begin{array}{*{20}l} y_{it} & = {\boldsymbol{x}^{\prime}}_{it} \beta + v_{it} - u_{it}, \end{array} $$

2 $$\begin{array}{*{20}l} v_{it} & \sim \mathcal{N}(0, \omega^{2}), \end{array} $$

3 $$\begin{array}{*{20}l} u_{it} & \sim \mathcal{N}^{+}(0, \sigma^{2}) \qquad i=1, \dots, N, \quad t=1, \dots, T. \end{array} $$

In (1), *y*_*it*_ is the log output and ***x***_*it*_ is a *K*-dimensional vector of the log input factors of region *i* at time *t*. Stochastic deviations from the health frontier are captured by *v*_*it*_, which is conventionally assumed to be normally distributed with zero mean and variance *ω*^2^. The inefficiency term *u*_*it*_ follows a half normal distribution with mean zero and variance *σ*^2^. To include demographic and socio-economic characteristics which might influence the individual inefficiency of health care provision in each region, the model fulfills the so-called scaling property [37]

4 $$ \sigma = \exp\left({\boldsymbol{z}^{\prime}}_{it} \delta\right).  $$

In (4), ***z***_*it*_ is a *R*-dimensional vector of the individual explanatory variables of the variance of the inefficiency term *u*_*it*_ for each country *j*. By virtue of the scaling property, the shape of the distribution of the inefficiencies *u*_*it*_ is the same for all regions [37]. This is intuitively appealing, as in general all regions have the same possibilities to reach the efficiency frontier. Demographic and socio-economic characteristics shape the deviations from the health frontier. The model in (1) - (4) is estimated by means of Maximum-Likelihood methods.

In efficiency analyses the main interest lies on the estimation of technical efficiency which is the ratio of the observed output and the maximum feasible output on the production frontier [8]. It is measured as

5 $$ \text{TE}_{it} = \frac{y_{it}}{\tilde{y}_{it}}= \frac{{\boldsymbol{x}^{\prime}}_{it} \beta + v_{it} - u_{it}}{{\boldsymbol{x}^{\prime}}_{it} \beta + v_{it}},  $$

where $\tilde {y}_{it}$ is the maximum feasible output which lies on the production frontier.

### Metafrontier model

The general (pooled) stochastic frontier model assumes homogeneous technologies for all individuals. In case of a comparison of health care systems across countries, this assumptions is far fetched. Even though, similar inputs and technologies are available to all the usage pattern, however, differs within the European countries due to different rules and regulations. A common production frontier cannot sufficiently account for these differences. In a metafrontier approach, it is possible to evaluate how efficient each country works in producing health and to compare the productivity and efficiency across nations without assuming similar technologies. The metafrontier approach is essentially a two step procedure developed by Battese et al. [4] and O’Donnell et al. [28]. In a first step, the group-specific (country-specific) frontiers are estimated by means of a stochastic frontier model as in (1) - (4) for each country separately. Accordingly, the estimated model parameters *β* and *δ* of the pooled SFA model in (1) and (4) change to *β*^*j*^ and *δ*^*j*^ for each country *j*. In a second step, a metafrontier is enveloped over the individual frontiers. Battese et al. [4] show that the metafrontier optimization can be solved by a linear programming problem for log-linear production functions according to

6 $$ \begin{aligned} \min_{\beta^{\ast}} \text{ }& L \equiv \sum_{i=1}^{N} \sum_{t=1}^{T}({\boldsymbol{x}^{\prime}}_{it}\beta^{\ast} - {\boldsymbol{x}^{\prime}}_{it}\hat{\beta}^{j}) \\ \text{s.t. }& {\boldsymbol{x}^{\prime}}_{it}\beta^{\ast} \geq {\boldsymbol{x}^{\prime}}_{it}\hat{\beta}^{j}. \end{aligned}  $$

In (6), *β*^∗^ is a vector of the metafrontier parameters and $\hat {\beta }^{j}$ is a vector of the estimated country specific stochastic frontier parameters. As the $\hat {\beta }^{j}$ are assumed to be fixed in the linear programming problem, (6) is equivalent to $\min _{\beta ^{\ast }}\text {} L \equiv {\boldsymbol {\bar {x}}^{\prime }}\beta ^{\ast }$, where $\boldsymbol {\bar {x}}$ is a vector of the means of all input variables for all observations [4].^1^ The standard errors for the metafrontier parameters can either be obtained by bootstrap or simulation.

To compare the efficiency scores of the countries across different technology sets (frontiers), the technical efficiency with respect to the metafrontier can be calculated according to

7 $$ \text{TE}_{it(j)}^{\ast} = \frac{y_{it(j)}}{y_{it}^{\ast}}= \frac{{\boldsymbol{x}^{\prime}}_{it(j)} \beta_{(j)} + v_{it(j)} - u_{it(j)}}{{\boldsymbol{x}^{\prime}}_{it} \beta^{\ast}},  $$

where $y_{it}^{\ast }$ is the output on the metafrontier. $TE_{it(j)}^{\ast }$ is the ratio of the observed output of region *i* at time *t* to the metafrontier output.

The ratio of the output of the prodcution function for country *j* relative to the potential output of the metafrontier for a given set of input variables is the metatechnology ratio (MTR) which is given by

8 $$ \text{MTR}_{it(j)} = \frac{\text{TE}_{it(j)}^{\ast}}{\text{TE}_{it(j)}}.  $$

**Fig. 1:**
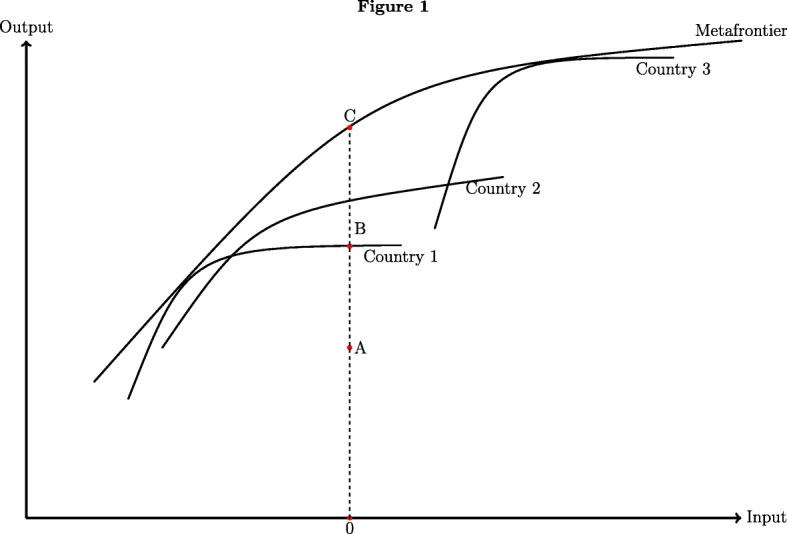
Metafrontier model. The graph shows the group specific frontiers for three groups (Country 1 to 3) and the metafrontier. Own presentation based on O’Donnell et al. [28]

The MTR captures the difference between the productivity between the group and the metatechnology (the technology available to all countries). Figure 1 illustrates the concept of the metafrontier graphically. The production model is set to a single input - single output framework with three convex country frontiers labelled Country 1, 2, and 3. The metafrontier envelopes the three country frontiers. It is assumed to be a deterministic smooth function with values no less than the individual country functions. Point A indicates a point of production of region *i* at time *t*. The figure illustrates the technical efficiency of region *i* with respect to its group frontier ($\overline {\text {0A}}/\overline {\text {0B}}$) and the respective technical efficiency $TE_{it}^{\ast }$ of region *i* to the metafrontier ($\overline {\text {0B}}/\overline {\text {0C}}$). Further, it shows the distance between the respective country’s production set and the metafrontier ($\overline {\text {0B}}/\overline {\text {0C}}$) [24, 28].

### Data and variables

Region-specific data on mortality, health care infrastructure and other characteristics are extracted from Eurostat online database.^2^ Annual data cover the period from 2005 to 2014. The panel is unbalanced due to the non-availability of data for certain regions and years. The overall number of observations in the study is 1149 (see Appendix Appendix A: Data description for a detailed description of the data). We base the regional analysis on the NUTS-2 (Nomenclature des unités territoriales statistiques) regions.

#### Health production

**Fig. 2:**
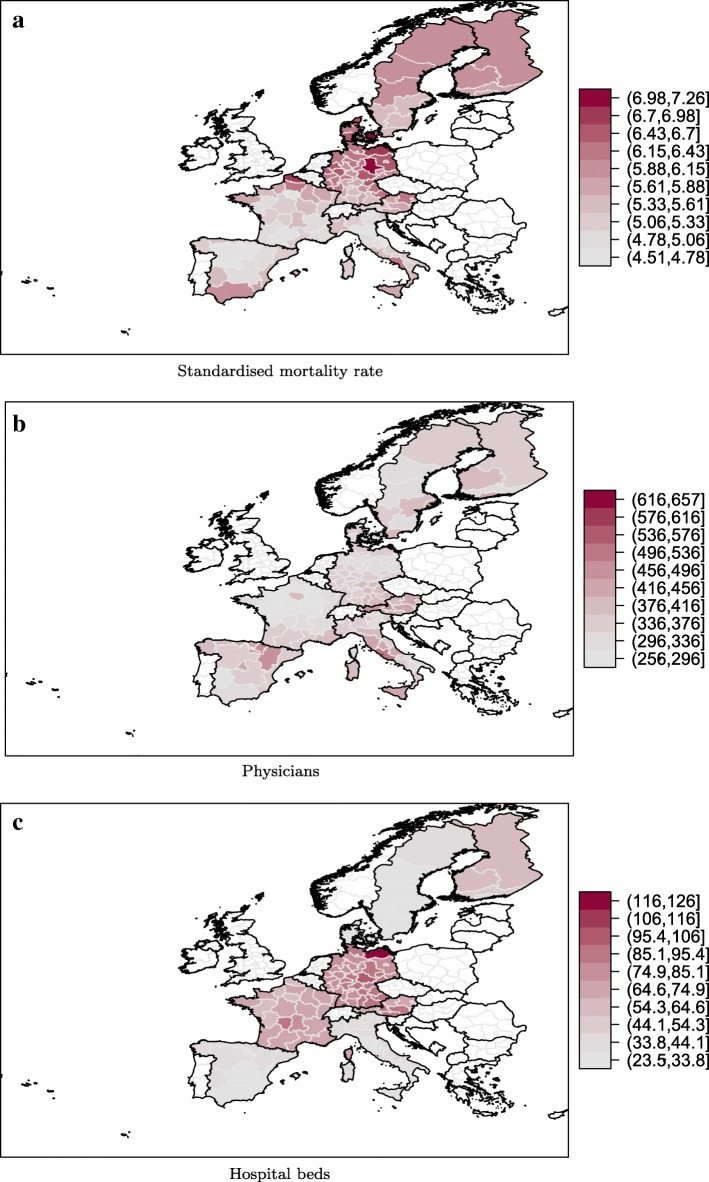
Health care output and inputs. The figure presents the spatial distribution for the average standardised mortality rate (**a**) and physician density per 100,000 inhabitants (**b**) and the number of hospital beds per 10,000 inhabitants (**c**)

It is hardly possible to directly measure the health of the population. The health status of the population can merely be approximated by measures such as life expectancy, mortality or morbidity. As specific measures of morbidity are not available at regional level, we use an age and sex standardised mortality rate (SMR) to resemble the population’s health status (see Appendix Appendix A: Data description for detailed information on the construction of the standardised mortality rate). The standardised mortality rate takes differences in the age and sex distribution of the population into account. By means of the standardisation, we calculate an indicator of the population’s health status that reflects the number of deaths that would have occurred if the European regions would have the same age and sex composition. To account for the health status of the population, we consider the inverse of the SMR. Panel (a) of Fig. 2 shows the spatial distribution of the average SMR. An visual inspections reveals a slight North-South gradient of the SMR for most countries with higher mortality rates in the South. In Italy and Spain the mortality rates appear to be lower in the South of the respective countries. Further, the SMR diverges between the regions in East and West Germany.

#### Health care infrastructure

The inclusion of health care inputs corresponds to the related literature [2, 6, 17, 23]. In our stylized specification of the country specific SFA model, we concentrate on the number of physicians (*doctors*) and the number of hospital beds (*beds*) both per 100,000 inhabitants as input variables representing the outpatient and inpatient sector, respectively as no data on the utilisation of health care services is available at such a high spatial resolution. Panels (b) and (c) of Fig. 2 display the average regional distribution of physicians and hospital beds, respectively. The spatial distribution of physicians does not exhibit a clear spatial pattern in Austria, Germany, Italy and Scandinavia. In France, the number of physicians is relatively low in the North. The area around Paris is an exception with a higher supply of physicians in comparison to the surrounding regions. The North of Spain is characterised by a higher supply of outpatient care. An eyeball inspection of Panel (c) reveals the highest densities of hospital beds in Finland. The supply of inpatient care exhibit a North-South gradient in Italy, Spain and Sweden with higher densities in the North of each country. Furthermore, the South of France and Austria and the North-East of Germany are characterised by slightly higher densities of hospital beds.

We further include the population density (*popdens*) to capture the degree of urbanisation of the region. Empirical evidence has shown a relationship between health and the population density of a region. However, a simple rural/urban differentiation does not sufficiently describe the relationship between health and location (see for instance Fassio et al. [16] and Adair [26]).

#### Socio-economic and demographic profiles

Besides the health care infrastructure demographic and socio-economic factors play an important role for the production of health and health outcomes [6]. In a health production framework those demographic and socio-economic factors can illustrate the utilisation of the health care infrastructure [20]. Due to varying regional utilisation structures, inefficiencies in the provision of health care services may arise. Possible sources of inefficiency include inaccurate and unnecessary medical treatments due to a lack of understanding and an over or under use of medical services [17, 20]. To control for different patterns in the utilisation of and the access to health care services we include the GDP per capita in (national) purchasing power parities, education, the share of the elderly and the population density in the inefficiency scaling function (as ***z***_*it*_ in (4)) similar to Herwartz and Schley [20]. The relationship between health and income is based on several factors. On the one side, income differences are directly related to differences in individual’s and population’s health status due to different conditions of life both at the individual and at the population level [25]. Empirical evidence has shown that regional (and individual) deprivation increases the risk of poor health [35]. As health care can be seen as a luxury good [30], regional deprivation does not only describe a direct link between health and income. Additionally, regional deprivation might account for access barriers to medical services [20]. We therefore include the GDP per capita (*gdp pc*) to describe regional deprivation.

The empirical literature has shown that a positive relationship between health and education exists [1, 12]. Higher education is related to a healthier life style which includes a healthy diet and exercises. Further, higher education is likely related to an improved understanding of medical treatments. To approximate education, we include the proportion of employees with a university degree as share of all employees (*education*). Both income and education can be seen to describe the access to and the utilisation of the regional health care services. To account for differences in the utilisation patterns associated with age, we include the share of the elderly (*age65*) as older age is related to an intensified need of medical treatment.

Additionally, we include the population density (*popdens*) in the inefficiency scaling function to control for factors influencing the efficiency of service production based on the location. As all countries offer a general coverage of the population by means of statutory health insurances or a taxed based National Health Services we do not control for price related access barriers.

Table 1 displays means, pooled standard deviations as well as within and between standard deviations. Cross-sectional heterogeneity across different NUTS 2 regions is more pronounced than the time heterogeneity. The stochastic frontier model for the production of health care in the European regions is estimated for all variables in the production function and *gdp p.c.* and *popdens* in logarithmic form.

**Table 1:** Descriptive Statistics. The table documents descriptive statistics for the 125 NUTS-2 regions from 2005 to 2014. The overall number of observations is 1149. In the second column the pooled sample means are reported, the third column contains the unbiased pooled standard deviations. In the last two columns the between and within sample standard deviations are presented, respectively

	Mean	SD	Between SD	Within SD
*SMR*	5.71	0.69	0.61	0.34
*doctors*	370.23	77.71	62.72	44.83
*beds*	600.19	236.59	235.98	29.12
*popdensity*	311.13	684.23	660.49	26.45
*gdp p.c.*	27647.00	6787.16	6556.47	1674.96
*education*	24.73	7.54	7.49	2.40
*age65*	0.19	0.03	0.03	0.01

## Results and discussion

In the following, we discuss estimation and inferential results for the group specific stochastic frontier models and the metafrontier model. First, we discuss the relationship between the health care infrastructure and overall health. Second, we examine the extend to which demographic and socio-economic characteristics shape deviations from the health frontier. Third, we analyse the regional distribution of efficiency scores and how efficiency levels change with respect to the metafrontier estimation. If not mentioned otherwise, the discussion of estimation results refers to the nominal 5% significance level.

### Elasticities of health care service provision

Table 2 documents the estimation results for the SFA model for pooled and group specific frontiers and the metafrontier model. Comparing the results of the group specific frontiers and the pooled model, which analyses the data for all countries simultaneously, shows that applying a joint model for all countries does not sufficiently capture the differences in health care production across the countries as the model parameters differ across models. To check the appropriateness of the metafrontier model, we apply a Likelihood Ratio (LR) test to test if technological differences between the countries are statistically significant.^3^ Particularly, the test statistic for the LR test is *LR*=1214.8 and *χ*^2^ distributed with 50 degrees of freedom. We can therefore reject the null hypothesis of identical group frontiers.

The results of the group frontiers indicate a positive relationship between the number of physicians and health care outcomes. Solely, the estimated effect for doctors in Italy is negative. Somewhat surprising, the number of hospital beds negatively relates to population’s health. This counter-intuitive effect might relate to an inappropriate distribution of health care infrastructure. Similarly, Herwartz and Schley [20] find a negative association between the supply of hospital beds and health outcomes in the German districts. Noticing the negative connection between inpatient care and health outcomes is in line with the supply-sensitivity of medical care. Accordingly, the supply and availability of medical resources influences its utilisation [36]. In other words, in regions with an increased level of inpatient care the hospital admission rates are relatively higher with likely adverse effects on health [36]. In line with this, Fisher et al. [18] find for the US that increased regional mortality rates are associated with a relatively high level of health care expenditure.

**Table 2:** Stochastic frontier and metafrontier estimation results (*t*-statistics in parentheses). This tables documents the estimation results from the regression model in (1) - (4) using data from 2005 to 2014. The second columns shows the regression results for a pooled model for all countries. The last column gives the metafrontier parameter results as in (6). The t-statistics for the metafrontier parameters are based on simulated standard errors (simulation with 500 replications)

	Pooled	Austria	France	Germany	Italy	Scandinavia	Spain	Metafrontier
*Output elasticities* (*x*_*it*_)								
Intercept	0.013	-0.042	0.034	0.01	0.053	-0.04	0.065	0.152
	(1.42)	(-3.63)	(4.18)	(1.62)	(5.68)	(-5.91)	(4.05)	(11.31)
ln(*doctors*)	0.092	0.261	0.465	0.219	-0.07	0.539	0.161	0.125
	(4.11)	(3.90)	(12.33)	(5.59)	(-2.44)	(11.31)	(4.54)	(3.46)
ln(*beds*)	-0.109	-0.275	-0.551	-0.258	-0.149	-0.213	-0.148	-0.209
	(-13.67)	(-4.91)	(-16.46)	(-5.75)	(-3.46)	(-8.62)	(-2.88)	(-6.99)
ln(*popdens*)	-0.006	-0.071	-0.049	-0.058	-0.034	-0.079	-0.016	-0.035
	(-1.47)	(-8.25)	(-6.53)	(-7.19)	(-3.17)	(-8.41)	(-2.21)	(-4.19)
*Effects on inefficiency* (*z*_*it*_)								
Intercept	-1.49	-0.134	3.421	-1.193	1.455	-8.408	1.119	
	(-1.87)	(-0.07)	(3.73)	(-1.36)	(1.69)	(-1.72)	(1.47)	
ln(*gdp p.c.*)	-1.83	-5.878	-2.449	-3.344	-1.38	-1.765	-1.056	
	(-4.2)	(-4.43)	(-2.97)	(-4.78)	(-3.48)	(-0.67)	(-1.21)	
*education*	-0.031	-0.019	-0.089	-0.128	-0.39	-0.139	-0.12	
	(-2.2)	(-0.34)	(-3.29)	(-6.04)	(-5.20)	(-2.10)	(-3.69)	
*age65*	-4.26	-25.972	-22.513	2.791	5.303	23.964	0.303	
	(-1.03)	(-2.60)	(-4.00)	(0.63)	(1.03)	(1.18)	(0.06)	
ln(*popdens*)	0.564	-0.937	0.343	-0.451	0.092	-1.954	0.212	
	(4.03)	(-1.65)	(1.73)	(-1.70)	(0.54)	(-4.00)	(2.16)	
*σ*_*v*_	0.994	0.932	0.953	0.943	0.966	0.924	0.984	
$\gamma =\sigma _{u}^{2}/\sigma ^{2}$	0.318	0.92	0.882	0.72	0.846	0.731	0.842	
log-likelihood	968.757	138.016	350.592	525.984	228.379	153.657	179.541	
no of observations	1149	90	218	368	177	116	180	

At first glance, the negative association of the population density and health seems counter-intuitive. However, as others have shown (see for instance [10, 16]) a low population density relates to an improved quality of life.

A note of caution is in order regarding the interpretation of the empirical results due to the potential of estimation bias as a result of reversed causality. For instance, more health care services could possibly be available in regions with a higher need, i.e. poorer population’s health. Nevertheless, the health care sector is a highly regulated market in which fundamental market mechanisms might fail [9]. For example, the regional planning of health care services is based on allocation formulas in Germany which do not or only implicitly take morbidity into account [20]. Moreover, it is noteworthy that regulators do not know mortality and morbidity rates at the time of structural planning weakening the potentials of endogeneity bias (see also [20]).

### The effect of socio-economic factors on the efficiency of health care services

In order to identify how the access to and the utilisation of the health care systems shapes the efficient provision of medical services, we examine the relationships of demographic and socio-economic variables and the efficiencies of health care service provision. Interestingly, many estimated coefficients in the medium panel of Table 2 are significant.

Income positively relates to the efficient provision of medical services. The estimated coefficients attached to *gdp p.c.* lack statistical significance in Scandinavia and Spain. This result is intuitively appealing as low income families are likely confronted with access barriers resulting in a lower utilisation of and satisfaction with the health care system [13]. Furthermore, we diagnose a positive relationship between the proportion of university graduates in the overall number of employees and efficiency. The effect lacks statistical significance only in Austria. Education helps to improve the execution of medical treatments and might enhance the utilisation of preventive care which might reduce inefficiencies in the health care sector. Additionally, as empirical literature has shown, people with higher educational achievements might have a lower burden of disease due to healthier lifestyle choices [1, 12]. Initially, the positive association of the number of senior citizens and the performance of health care systems in Austria and France seems somewhat surprising. One would expect that more co-morbidities linked with old age would likely decrease the efficient provision of health care services. Similarly, Eibich and Ziebarth [14] find a direct positive correlation between the share of the elderly and well-being at the individual level possibly related to the provision of improved health care resources in regions with an older population.

The relationship between the population density and inefficiency depends on the specified country frontier. We diagnose a negative association between efficiency and the population density in the pooled model and the model for Spain. In Scandinavia, the relationship is reversed. The effects lack significance for all other countries.

Taken together, the results provide important insights into how demographic and socio-economic factors shape the efficient provision of health care services. The results highlight that allocation rules for medical infrastructure should take those factors into account (see for instance Smith [34] for the case of the UK). Furthermore, reducing access barriers possibly increases the efficiency of health care service provision by promoting the utilisation of preventive care. Additionally, raising the awareness in health care personnel (i.e. physicians and nurses) for the needs of specific (deprived) population groups likely decreases inefficiency by possibly improving the communication leading to a better understanding of medical treatments (see also Herwartz and Schley [20]).

### Metafrontier estimates

The different parameter estimates in the group specific frontiers indicate that differences in the production technology of the respective health care systems exist. To investigate if those country specifics trigger differences in the efficiency of the health care provision, we analyse the health care systems by means of a metafrontier approach. The last row of Table 2 reports the parameter estimates and *t*-statistics based on simulated standard errors (see Battese et al. [4]). In line with the parameter estimates attached to the elasticities of the pooled model and the group frontiers, the results reveal a positive association of physicians and health. We find a negative relationship between hospital beds and the populations density and health.

### Regional distribution of efficiency scores

Table 3 documents the average efficiency scores^4^ for the pooled model, the country-specific frontiers, the MTR and the technical efficiencies with respect to the metafrontier. Note that the efficiency scores of the country-specific frontiers cannot be compared across groups as they are calculated with respect to different technologies [28].

**Fig. 3:**
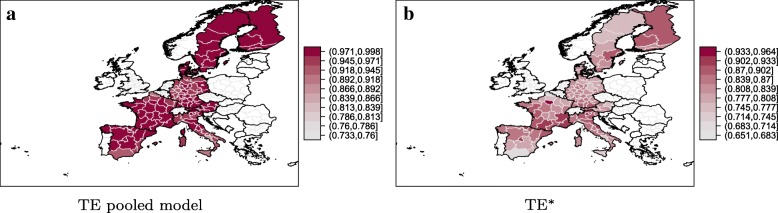
Spatial distribution of the average technical efficiency scores for the pooled model (**a**) and the TE^∗^ with respect to the metafrontier (**b**)

For the sampled countries, the technical efficiency scores for the pooled model range from 0.6518 in Ciudad Autónoma de Ceuta, Spain and 0.9985 in Övre Norrland, Sweden with an overall average of 0.9696. In the pooled model the regions in Scandinavia perform the best while the Italian regions exhibit relatively low efficiency scores. These results are intuitively appealing as the Scandinavia social security systems enjoy an excellent reputation. Panel (a) of Fig. 3 shows the regional distribution of the average technical efficiency scores of the pooled model. A visual inspection reveals a limited regional variation of efficiency across countries.

**Table 3:** Technical efficiency (TE) and metatechnology ratio (MTR) for group frontiers and metafrontier. This table documents the average technical efficiencies for the respective countries for the pooled model in the first column, the average efficiencies for the group specific models in the second column, the average MTR in the third column and the average TE with respect to the metafrontier (TE^∗^) in the rightmost column for 2005 to 2014

	TE	TE	MTR	TE^∗^
	(pooled model)	(country specific)		
Austria	0.9700	0.9403	0.8668	0.8146
France	0.9746	0.9461	0.8938	0.8462
Germany	0.9669	0.9577	0.8785	0.8412
Italy	0.9583	0.9377	0.9097	0.8552
Scandinavia	0.9867	0.9662	0.8563	0.8249
*Denmark*	0.9725	0.9997	0.7883	0.7881
*Finland*	0.9933	0.9904	0.8883	0.8795
*Sweden*	0.9911	0.9421	0.8772	0.8234
Spain	0.9592	0.9207	0.8959	0.8244

The average efficiency scores of the country-specific frontiers are slightly lower compared to the pooled model. The relatively high group-specific efficiencies indicate that the regional health care systems use the available resources efficiently.

In the next step, we compare the MTR and the TE scores with respect to the metafrontier (TE^∗^). The MTR measures how close the country-specific frontier is to the metafrontier. Higher (lower) values of the MTR imply a smaller (larger) technology gap between the country specific individual frontier and the metafrontier. The MTR ranges from 0.7071 (Sjælland, Denmark) to unity (Övre Norrland in Denmark, Länsi-Suomi in Finland, and Basilicata in Italy). The MTR values equal to unity indicate that the individual country frontiers are tangent to the metafrontier. The *TE*^∗^ range from 0.5719 in Ciudad Autónoma de Ceuta, Spain to 0.9979 in Länsi-Suomi, Finland. The overall mean is 0.8375. Panel (b) of Fig. 3 displays the regional distribution of the efficiency scores with respect to the metafrontier. The graph highlights some interesting regional pattern with higher efficiencies in the North of Finland, Italy and Spain and the South of France and Germany. Somewhat surprisingly, the MTR results hint at a large technology gap in Scandinavia. In combination with high country-specific efficiency scores, the results indicate that the regions in Scandinavia would profit from rising the production potentials as the productivity with respect to their own frontier is already very high. Similar to the results of others (see for instance Joumard and Nicq [22]) this might highlight failing market mechanisms within a highly regulated health care system. For Italy and Spain, which on average perform the worst with respect to the pooled (and country specific) frontiers, the results imply that a better management of and an improved access to and utilisation of the available resources could likely lead to efficiency gains.

One can interpret the metafrontier as highlighting long-run production potentials [27]. Accordingly, the relatively low TE ^∗^ hint at substantial scopes of improvement in the regional provision of health care services in Europe.

## Conclusion

The health care systems around Europe are faced by challenges regarding the demographic change along with expensive medical innovations. In regard of those developments an efficient use of the scarce financial resources is necessary. Based on the different health care systems in Europe, the European governments adopt different strategies to deal with this and to guarantee an efficient use of the financial resources.

This paper analyses the efficiency of health service provision across several European countries by means of a stochastic metafrontier approach. The application of a metafrontier has the advantage that it is possible to distinguish between regional efficiency in relation to the country’s own frontier and an European metafrontier.

The results show that a single European stochastic frontier model cannot sufficiently capture the heterogeneous conditions of health care provision in Europe. The comparison of the spatial distribution of the efficiency scores from a pooled European model assuming a homogeneous technology hints at significant efficiency differences across countries. The Scandinavian countries achieve on average the highest efficiency scores in a pooled model. Surprisingly, the regions in Scandinavia lag behind other regions with respect to the metafrontier highlighting potentials for rising the productivity in these regions by, for instance, easing some regulatory burdens. The relatively lower efficiency scores in Italy and Spain hint at substantial opportunities to rise the populations’ health by improving the management of and the access to the available resources.

## Appendix A: Data description

### Sample

The data cover the period 2005 to 2014. We analyse the health care systems at the NUTS-2 (Nomenclature des unités territoriales statistiques). The data is mainly drawn from the Eurostat database. For Germany, the infrastructural variables were not available at NUTS level. We approximate the number of hospital beds and physicians by the respective number of the federal states.

### Standardised mortality rate

Annual data on sex and age specific number of deaths and inhabitants on district level are provided by the Eurostat online data base. As the regions’ population differ in age and sex composition we use the direct standardisation to obtain a standardised mortality rate. The mortality rate then represents the expected number of deaths if the age and sex structures in all districts were identical. We use the European standard population 2013 for the standardisation.

### Health care infrastructure

The number of physicians and hospital beds are provided by Eurostat. Both variables are measured per 100.000 inhabitants.

### Socio-economic and demographic characteristics

The data on socio-economic and demographic characteristics are drawn from the Eurostat data base. The population density is the number of inhabitants per 1.000m^2^. The gdp p.c. is the regional gdp per capita in (national) purchasing power parities. Education is approximated by the share of employees with a university degree. We calculate the share of the elderly based on the population statistics provided by Eurostat.

## Abbreviations

LR: Likelihood ratio
MTR: Metatechnology ratio
NUTS-2: Nomenclature des unité
s territoriales statistiques; SFA: Stochastic frontier analysis
SMR: Standardised mortality ratio
TE: Technical efficiency
WHO: World Health Organisation

## Footnotes

1 For a more detailed discussion of the minimization problem in (6) see Battese et al. [4] and O’Donnell et al. [28].

2 For Germany, we approximate the number of physicians and hospital beds by the respective number on federal state level as no information on NUTS-2 level is available.

3 The test statistic for the LR test is *L**R*=−2[ln(*L*(*H*0))−ln(*L*(*H*1))], where ln(*L*(*H*0)) is the log-likelihood value of a stochastic frontier model estimated by pooling the data for all countries, and ln(*L*(*H*1)) is the sum of the log-likelihood values of the country specific stochastic frontier models. The test statistic is *χ*^2^ distributed. The degrees of freedom are the difference between the number of parameters estimated in the individual stochastic frontier models and the number of parameters estimated in the pooled model.

4 The point estimates of technical efficiency for region *i* at time *t* are defined according to Battese and Coelli [3] as TE_*it*_=*E*(*e**x**p*(−*u*_*it*_|*ε*_*it*_) where *ε*_*it*_ is the composite error term of the production function, i.e. *ε*_*it*_=*v*_*it*_−*u*_*it*_.
